# Porous organic polymer with *in situ* generated palladium nanoparticles as a phase-transfer catalyst for Sonogashira cross-coupling reaction in water†

**DOI:** 10.1039/c9ra04103f

**Published:** 2019-07-12

**Authors:** Ying Dong, Yun-Qi Chen, Jing-Jing Jv, Yue Li, Wen-Han Li, Yu-Bin Dong

**Affiliations:** College of Chemistry, Chemical Engineering and Materials Science, Collaborative Innovation Centre of Functionalized Probes for Chemical Imaging in Universities of Shandong, Key Laboratory of Molecular and Nano Probes, Ministry of Education, Shandong Normal University Jinan 250014 P. R. China dongyinggreat@163.com yubindong@sdnu.edu.cn

## Abstract

A new Pd nanoparticle loaded and imidazolium-ionic liquid decorated organic polymer of Pd@PTC-POP was readily fabricated *via* a Pd(PPh_3_)_4_ catalysed *in situ* one-pot Suzuki cross-coupling reaction between imidazolium attached dibromobenzene and 1,3,5-tri(4-pinacholatoborolanephenyl)benzene. Besides the high thermal and chemical stability, the obtained Pd@PTC-POP can be used as a highly active and reusable phase-transfer solid catalyst to promote the Sonogashira coupling reaction in water. The obtained results indicate that the Pd@PTC-POP herein could create a versatile family of solid phase transfer catalysts for promoting a broad scope of reactions carried out in water.

## Abstract 2

A Pd nanoparticle loaded and imidazolium-ionic liquid decorated organic polymer, which can be used as a highly active phase-transfer solid catalyst to promote the Sonogashira reaction in water, was reported.

## Graphical abstract

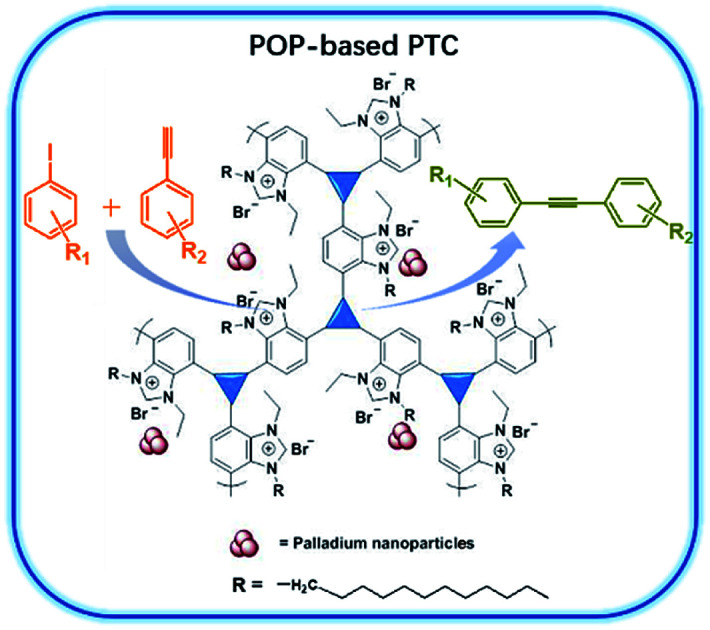

## Introduction

Phase-transfer catalysis (PTC), which can accelerate an aqueous phase-organic phase reaction, has attracted more and more attention recently due to its eco-friendly and low-cost solvent system, mild reaction conditions and relatively simple operating procedure.^1^ However, some congenital disadvantages of the conventional PTC, such as difficulty in the separation and recycling of the surfactant catalysts (*i.e.*, quaternary ammonium salts and so on),^2^ seriously limited its practical applications. The solid-supported PTC technique might be an alternative approach for addressing these issues, because it is more favourable for PT catalyst isolation and recovery, consequently leading to a cleaner and greener catalytic process.^3^

As is known, Pd nanoparticles (Pd NPs) are highly active and have been widely used in promoting carbon–carbon cross-coupling reactions,^4^ However, they are prone to aggregating and forming Pd black because of their high surface energy.^5^ For addressing this issue, Pd NPs are usually immobilized in porous supports such as zeolites,^6^ metal oxides,^7^ metal–organic frameworks (MOFs)^8^ and covalent organic frameworks (COFs).^9^

Porous organic polymers (POPs), as a typical class of porous organic material, are an additional important class of solid support to upload and stabilize metal NPs.^10^ On the other hand, surfactant groups like imidazolium-based ionic liquid (IM-IL) could be readily introduced into POPs by combination of the pre-modified organic building blocks.^11^ In doing so, the Pd NP catalytic functionality, IM-IL PTC property and POP-based heterogeneous catalytic nature would be perfectly integrated together to lead to multifunctional catalytic systems which can eventually meet the requirements of sustainable chemistry and green synthesis.

In this contribution, for the first time, we report a Pd NP loaded and IM-IL decorated POP material *via* an *in situ* one-pot synthetic approach, and the obtained Pd@PTC-POP with long *n*-dodecyl chains can be used as a highly active solid phase-transfer catalyst to promote the Sonogashira reaction in water.

## Results and discussion

### Synthesis and characterization of Pd@PTC-POP

**Scheme 1:**
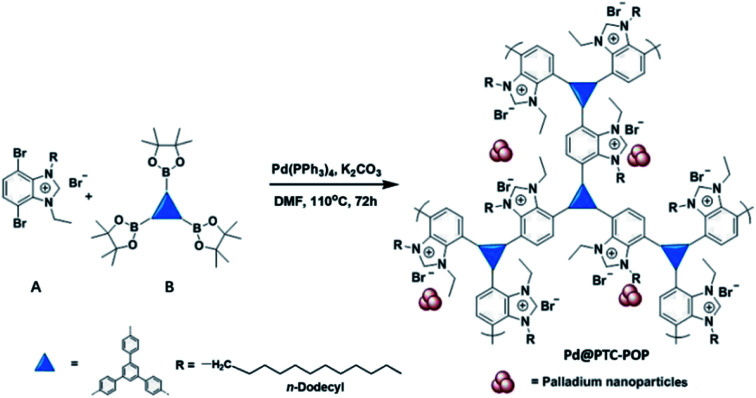
Synthesis of Pd@PTC-POP*via in situ* one-pot synthetic approach.

As shown in Scheme 1, Pd@PTC-POP was readily prepared by combination of the long *n*-dodecyl chain decorated A and 1,3,5-tri(4-pinacholatoborolanephenyl)benzene B by Pd-catalysed Suzuki–Miyaura reaction in DMF at 110 °C. IR spectra showed that the characteristic peaks at 1359 cm^−1^ and 622 cm^−1^ attributed to the B–O in B and C–Br in A species significantly decreased after coupling reaction, meanwhile the bands at *ca.* 2923 and 2852 cm^−1^ that corresponded to the alkyl groups appeared, indicating that the precursors of A and B in Pd@PTC-POP are successfully connected to each other *via* C–C binding interaction (Fig. S1, ESI†). In addition, the solid-state ^13^C NMR showed that the peaks at 140, 138 and 126 ppm are associated with the phenyl and imidazolyl carbons. The broad signals at 41–38, 28.5, 21.9, 12.9 ppm were ascribed to the aliphatic carbon atoms from precursor of A (Fig. S1, ESI†). The thermal stability of the resultant Pd@PTC-POP and the degree of lost has been investigated by thermogravimetric analysis (TGA) and differential thermal gravity (DTG), as shown in Fig. S1.† One sharp breakdown of percentage weight loss was observed at 280 °C. Two flat breakdowns of percentage weight loss were observed at 350 and 520 °C respectively, in the TGA profile. Before 220 °C, the total 3% weight loss should be attributed to the evaporation entrapped DMF. The temperature range of 280–350 °C might correspond to the degradation of the Pd@PTC-POP into removable gases like CO_2_ and NO_2_. No substantial weight loss at higher temperature above of 550 °C, representative of decomposition of all the organic species, was completed.^12^ The Pd@PTC-POP was insoluble in water and common organic solvents, which is the typical feature of polymeric organic species.

**Fig. 1:**
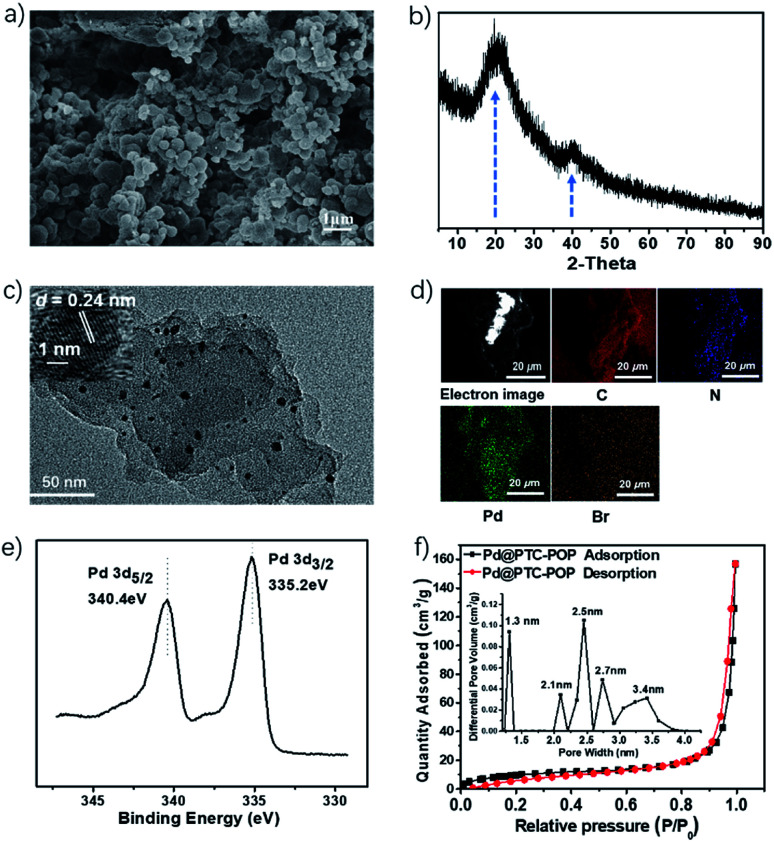
SEM image (a), PXRD patterns (b), HRTEM image (c), SEM image and elemental mapping (d), XPS spectrum of the encapsulated Pd species (e) and N_2_ sorption isotherm of Pd@PTC-POP (f). The pore size distribution plot of Pd@PTC-POP is shown as the inset. Several sharp peaks at 1.3, 2.1, 2.5 and 2.7 nm and one broad peak at 3.4 nm were observed, which are attributed to the existence of micropores and interparticle mesopores throughout the entire POP matrix.

The obtained Pd@PTC-POP was the irregular granular particle which was well evidenced by the scanning electron microscopy (Fig. 1a). Notably, the Pd NP in POP was *in situ* generated and trapped by the POP during the preparing process. As shown in Fig. 1b, the PXRD measurement exhibited a major broad peak centred at 20°, suggesting the amorphous nature of Pd@PTC-POP. Meanwhile, the weak peak at 40° was indexed to Pd (111) reflection, corresponding to the face-centred cubic (fcc) lattice arrangement of Pd(0) nanoparticles. Because of the very low Pd loading, the diffraction peaks of Pd (200), Pd (220) reflections cannot be obviously observed.^12^ The existence of Pd NPs was unambiguously confirmed by the high-resolution transmission electron microscopy (HRTEM). As indicated in Fig. 1c, the Pd NPs (2–6 nm) were homogeneously distributed in the POP matrix, and the atomic lattice fringes with an interplanar spacing of 0.24 nm corresponding to the 1/3 (422) fringes of face-centred cubic (fcc) Pd NP were clearly observed.^13^ The uniform texture of Pd@PTC-POP was further confirmed by the SEM-energy dispersive X-ray (EDX) mapping, which showed a homogeneous distribution of C, N, Pd, and Br elements in Pd@PTC-POP (Fig. 1d). Besides TEM, the oxidation state of the encapsulated Pd species was further examined by X-ray photoelectron spectroscopy (XPS) (Fig. 1e). The observation of Pd d_5/2_ and d_3/2_ peaks at 335.2 and 340.4 eV in the XPS spectrum of Pd@PTC-POP demonstrated the palladium to exist as Pd(0).^14^ Inductively coupled plasma (ICP) analysis showed that the palladium content in Pd@PTC-POP was 0.547 wt%.

The N_2_ sorption isotherm was measured at 77 K to characterize the specific surface area with architectural rigidity and permanent porosity of Pd@PTC-POP (Fig. 1f). Brunauer–Emmett–Teller (BET) analysis showed that it featured a combination of type II and IV isotherms with a surface area of 37.7 m^2^ g^−1^ (Fig. 1f). It was suggested that the weak hysteresis of the isotherm is attributed to the swelling-ability of the polymer in condensed nitrogen. The encapsulated trace of palladium in POP might be responsible for this slight hysteresis, which was observed in the previous report.^15^ The low surface area of Pd@PTC-POP herein should be caused by the decorated long *n*-dodecyl chains. For confirm this, no long IM-IL-decorated Pd@POP was prepared by the combination of 4,7-dibromo-1-ethyl-1*H*-benzo[*d*]imidazole and B*via* the same Pd-catalysed Suzuki–Miyaura cross-coupling reaction (Fig. S2, ESI†). The *S*_BET_ of Pd@POP based on its N_2_ sorption isotherm at 77 K was found to be 332 m^2^ g^−1^, so the attached IM-IL moiety on POP resulted in an 89% surface area decrease.^16^

### Catalytic property

By taking advantage of the decorated long IM-IL species, together with the embedded highly active Pd NP, we assumed that the obtained Pd@PTC-POP should be an ideal phase-transfer catalyst and could promote organic reactions in aqueous medium. Its catalytic performance was then evaluated using Sonogashira cross-coupling reaction between iodobenzene with phenylacetylene in water. As it is known, the Sonogashira cross-coupling reactions have been widely employed in synthesis of various important organic species such as pharmaceuticals, natural products, organic materials, and nanomaterials, but they were mostly performed in organic or organic-aqueous mixed media instead of pure water.^17^ Thus, the development of Pd-catalysed aqueous Sonogashira reaction would meet the increasing environmental and sustainable requirements.

Optimization of the reaction was first conducted with different base such as Cs_2_CO_3_, K_2_CO_3_ and Et_3_N to furnish the desired cross-coupling product of diphenylacetylene under the given reaction conditions. As shown in Table 1, the organic base Et_3_N (92% yield) was found to be a superior over the inorganic bases of Cs_2_CO_3_ (82% yield) and K_2_CO_3_ (28% yield) (entries 1–3). In addition, when the reaction was carried out in water with a higher catalyst loading, 0.3 mol% instead of 0.15 mol%, the coupled product was isolated in a significantly higher 99% yield (Table 1, entry 4). At 0.3 mol% Pd loading, the reaction time was dramatically shortened, but with the ideal isolated yields of 96% at 1 h (Table 1, entry 5) and 99% at 2 h (Table 1, entry 6), respectively. On the other hand, the reaction temperature appeared to be crucial to the catalytic efficiency. As indicated in Table 1, the catalytic activity of Pd@PTC-POP was largely diminished at lower temperature, only 5% yield was achieved when the reaction was performed at 60 °C (Table 1, entry 7). Also, the lower amount of base or phenylacetylene would lead to a significantly reduced yield under the given conditions. For example, when the reaction was carried out with 1.0 eq. or 2.0 eq. base, the product yields were obtained in 40 (Table 1, entry 8) and 70% yields (Table 1, entry 9), respectively. Furthermore, when 1.2 eq. instead of 2.0 eq. of phenylacetylene was employed, the coupled product was generated in 78% yield (Table 1, entry 10). Notably, the Pd-free PTC-POP (obtained by treatment of Pd@PTC-POP with HNO_3_) was also used to conducted the reaction (Table 1, entry 11), and no desired product was obtained, indicating that the loaded Pd NP was the catalytic active species (Fig. S3, ESI†). The turnover number (TON) and turnover frequency (TOF) for the model reaction under the optimized conditions (Pd 0.3 mol%, 2 h, 100 °C, NEt_3_, H_2_O) are 330 and 165 h^−1^, respectively.

**Table 1:** Optimization of the model Sonogashira coupling reaction between iodobenzene and phenylacetylene^a^

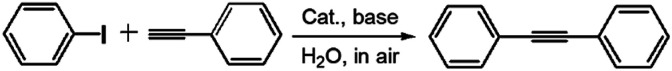						
Entry	Catalyst	Base	Pd (mol%)	*t* (h)	*T* (°C)	Yield^b^ (%)
1	Pd@PTC-POP	Cs_2_CO_3_	0.15	12	100	82
2	Pd@PTC-POP	K_2_CO_3_	0.15	12	100	28
3	Pd@PTC-POP	Et_3_N	0.15	12	100	92
4	Pd@PTC-POP	Et_3_N	0.3	12	100	99
5	Pd@PTC-POP	Et_3_N	0.3	1	100	96
6	Pd@PTC-POP	**Et**_**3**_ **N**	**0.3**	**2**	**100**	**99**
7	Pd@PTC-POP	Et_3_N	0.3	2	60	5
8^c^	Pd@PTC-POP	Et_3_N	0.3	2	100	40
9^d^	Pd@PTC-POP	Et_3_N	0.3	2	100	70
10^e^	Pd@PTC-POP	Et_3_N	0.3	2	100	78
11	PTC-POP	Et_3_N	—	2	100	—f
12	Pd@POP	Et_3_N	0.3	2	100	33

a Reaction conditions: iodobenzene (0.5 mmol), phenylacetylene (1.0 mmol), base (1.5 mmol, 3.0 eq. with respected to iodobenzene), H_2_O (3 mL), under air atmosphere.

b Isolated yield.

c 1.0 eq. Et_3_N.

d 2.0 eq. Et_3_N.

e 1.2 eq. phenylacetylene.

f Yield was determined by GC analysis (Fig. S3, ESI).

For further demonstrated the PTC functionality of Pd@PTC-POP, the catalytic activity of no IM-IL-decorated Pd@POP for the model Sonogashira cross-coupling between iodobenzene and phenylacetylene in water was also examined under the optimized conditions. As shown in Table 1 (entry 12), the isolated yield for the desired diphenylacetylene (3a) was only 33%, indicating that the IM-IL species in Pd@PTC-POP indeed played a key role in this Pd-catalysed PTC process.

**Fig. 2:**
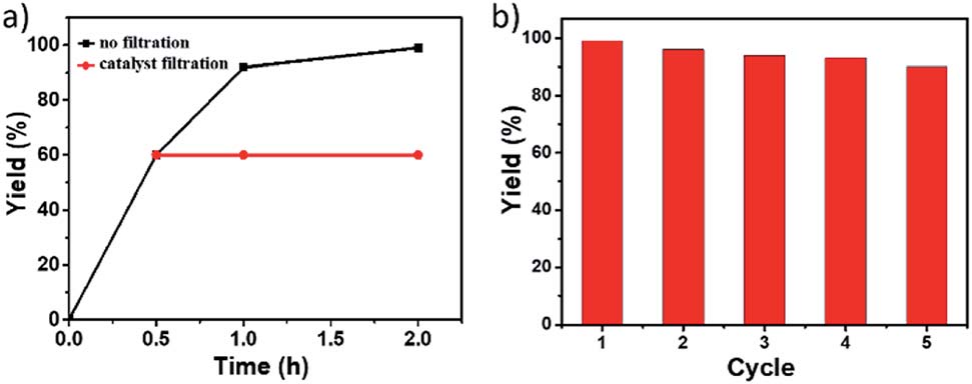
(a) Reaction time examination and leaching test for the model Sonogashira cross-coupling reaction. (b) Yield of product diphenylacetylene in repeated runs for the model Sonogashira cross-coupling reaction.

To gain insight into the heterogeneous nature of Pd@PTC-POP, the hot leaching test was conducted. As shown in Fig. 2a, no further reaction occurred without Pd@PTC-POP after ignition of the reaction at 0.5 h, indicating that Pd@PTC-POP exhibited a typical heterogeneous catalyst nature herein.

As a heterogeneous catalyst, its reusability was also examined. After each catalytic run, the solid catalyst was retrieved by centrifugation, washed with EtOH (3 × 2 mL), CH_2_Cl_2_ (3 × 2 mL), and dried at 110 °C for 2 h and then was reused for the next catalytic run under the same reaction conditions. As shown in Fig. 2b, the solid catalyst of Pd@PTC-POP still showed excellent activity and the cross-coupling yield was even up to 90% after five catalytic cycles. After multiple catalytic cycles, no obvious Pd NP aggregation occurred (Fig. S4, ESI†). The Pd amount in Pd@PTC-POP was 0.486 wt% (determined by ICP), suggesting *ca.* 11% Pd leaching occurred during reusable processes, which could be the reason for this slight yield drop. On the other hand, no valence change for Pd species was observed (Fig. S4, ESI†), implying that the Pd species in POP was stable during the reusable process. In addition, the POP morphology and elemental distribution were well maintained after the recycle (Fig. S4, ESI†). The slight shift for Pd(0) peaks in the XPS spectrum after five catalytic runs (ESI) should be caused by the tiny amount of Pd(0) oxidation during the reusable process because the catalytic reaction was performed in air at 100 °C.

**Fig. 3:**
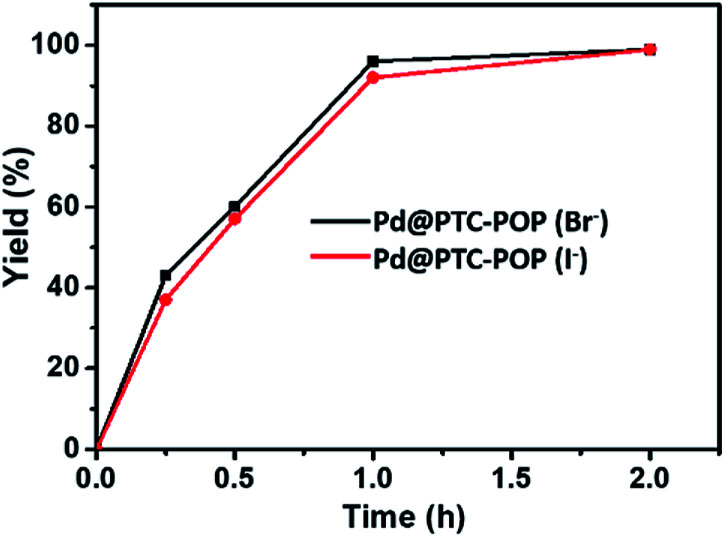
Comparison of catalytic activity of Pd@PTC-POP (Br^−^) and Pd@PTC-POP (I^−^) for the model Sonogashira coupling reaction between iodobenzene and phenylacetylene under the optimized conditions.

It is noteworthy that the Br^−^ in POP was largely replaced by I^−^ (79% based on elemental analysis) after the reusable process, however, the different X^−^ in POP herein did not affect the catalytic activity of Pd@PTC-POP for the model coupling reaction. For example, the model coupling reaction catalysed by Pd@PTC-POP with I^−^ also afforded product in 99% yield within 2 h under the optimized conditions (Fig. 3).

The excellent catalytic activity in water of Pd@PTC-POP encouraged us to further explore the generality of the catalytic system, and a series of substituted aryl iodides or arynes with wide range of functional groups such as –CO_2_Me, –CN, –NO_2_, –CF_3_, –Me and –OMe at different positions were tested under the optimized reaction conditions (Table 2). As shown in Table 2, aryl iodides with both electron-donating and electron-withdrawing groups at para- or ortho-substituted position afforded cross-coupling products (3a–g) with excellent isolated yields (93–99%, Table 2, entries 1–7). However, the iodobenzene with meta-substituted electron-donating group like 3-methyliodobenzene provided the coupled product of 3h in slightly lower 88% yield (Table 2, entry 8). For substituted arynes, the coupling yields for the substrates with electron-withdrawing and electron-donating group provided excellent 96–98% yields (Table 2, entries 9–11, 3i–k). In contrast, 3-chlorophenylacetylene, however, gave an 85% yield for the desired product of 3l, which was caused by the low activity of 3-chlorophenylacetylene, meanwhile 13% substrate was recycled after reaction (Table 2, entry 12). In addition, the coupling reactions on the both substituted aryl iodides and arynes with either electron-withdrawing or electron-donating groups were also carried out, they all afforded good-to-excellent yields ranging from 86–95% for 3m–o (Table 2, entries 13–15).

**Table 2:** Sonogashira cross-coupling reactions of various aryl iodides with arynes catalysed by Pd@PTC-POP^a^

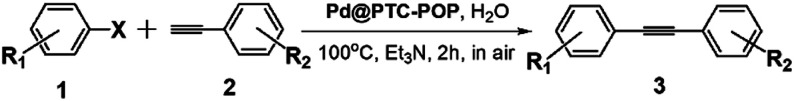					
Entry	R_1_	X	R_2_	Product	Yield^b^ (%)
1	H	I	H	3a	99
2	4-CN	I	H	3b	99
3	4-OCH_3_	I	H	3c	96
4	3-NO_2_	I	H	3d	93
5	2-CF_3_	I	H	3e	99
6	2-OCH_3_	I	H	3f	93
7	4-COOCH_3_	I	H	3g	99
8	3-CH_3_	I	H	3h	88
9	H	I	4-OCH_3_	3i	98
10	H	I	4-NO_2_	3j	97
11	H	I	3-CH_3_	3k	96
12	H	I	3-Cl	3l	85
13	4-CN	I	4-NO_2_	3m	91
14	2-CF_3_	I	4-OCH_3_	3n	95
15	2-OCH_3_	I	4-OCH_3_	3o	86
16	4-Ph	I	4-Ph	3p	46
17	H	Br	H	3a	72
18	4-NO_2_	Br	H	3j	85
19	4-OCH_3_	Br	H	3c	19
20	4-NO_2_	Cl	H	3j	27
21	H	Cl	H	3a	<5
22	4-OCH_3_	Cl	H	3c	—

a Reaction conditions: iodobenzene (0.5 mmol), phenylacetylene (1.0 mmol), Et_3_N (1.5 mmol), catalyst (0.3 mol% Pd), H_2_O (3 mL).

b Isolated yield (ESI).

In addition, a larger sized 4-phenyl iodobenzene and 4-ethynyl-1,1′-biphenyl were also used as the substrates to perform this Sonogashira coupling reaction under the same conditions (Table 2, entry 16). The coupling product of 3p was isolated in moderate 46% yield, which might result from the relatively slow diffusion of the large-sized substrates and product in the POP, moreover, suggesting that the Pd NPs are mainly located in the POP matrix instead of the surface, and the coupling reaction herein could be an internal surface catalytic process.

Besides iodo-substituted aromatic substrates, we also tested the catalytic activity of Pd@PTC-POP for the coupling reactions based on bromo- and chloro-substituted substrates. As shown in Table 2 (entry 17), the bromobenzene and phenylacetylene coupling under the given conditions provided the diphenylacetylene (3a) in 72% yield, suggesting that the bromo-substituted substrates were less reactive than those of corresponding iodo-substituted aromatics. It was similar to iodio-substituted substrates, the bromo-substituted benzene with the electron-withdrawing group proved effective coupling partner than that of bromobenzene with the electron-donating one. As indicated in Table 2, 4-nitrobromobenzene and 4-methoxybromobenzene furnished the desired products 3j (entry 18) and 3c (entry 19) in 85% and 19% yield, respectively. In contrast, chloro-substituted substrates showed even less reactivity toward the coupling reaction, but the same substituent effect was observed. As indicated in Table 2, the coupling of 4-nitrochlorobenzene with phenylacetylene gave the desired product 3j in 27% yield (entry 20), while using chlorobenzene provided corresponding product 3a in only <5% yield (entry 21). No desired product of 3c was isolated from the reaction of 4-methoxychlorobenzene and phenylacetylene under the given reaction conditions (Table 2, entry 22).

**Scheme 2:**
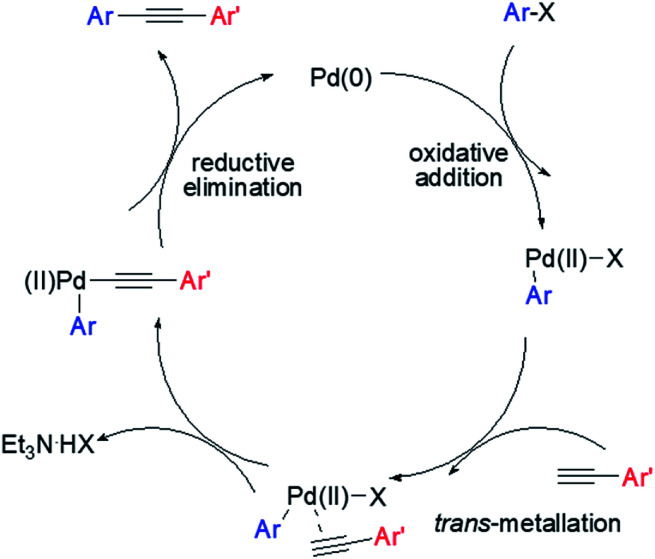
Proposed mechanism for the copper-free Sonogashira coupling reaction catalysed by Pd@PTC-POP.

The proposed mechanism of the Pd@PTC-POP catalysed Sonogashira cross-coupling reaction herein was shown in Scheme 2, which was believed to be the same as those of reported copper-free Sonogashira coupling reactions.^18^ Initially, oxidative addition of the aryl iodide to Pd(0) occurred. The alkyne subsequently went through an insertion and deprotonation processes. The formed intermediate further underwent a halogen displacement followed by a reductive elimination to give the coupled product.

Data on the reaction conditions, activity, and efficiency of the reported Pd-loaded heterogeneous catalytic systems employed earlier for the Sonogashira cross-coupling of aryl iodides with phenylacetylene are given in Table 3. Comparison of the results indicated that the Pd@PTC-POP herein met the green synthesis and sustainable requirements such as pure water reaction medium, cyclic utilization and high catalytic efficiency, which made it in a strong position among the reported catalysts.

**Table 3:** Comparison of Pd@PTC-POP with the typical reported Pd-loaded solid catalysts for the Sonogashira cross-coupling reaction between iodobenzene and phenylacetylene

Cat. (mol%)	Cu	Conditions	Yield (%)	Run	TOF (h^−1^)	Ref.
Pd@Hal-CS-SFIL (10)	✗	2 h, K_2_CO_3_, 90 °C, EtOH	96	7	4.8	19
Pd@Hal-PAMAM-G1-ISA (0.015)	✗	1.25 h, K_2_CO_3_, 65 °C, H_2_O/EtOH	97	10	554	20
OxPdCy@clay (0.05)	✗	24 h, K_2_CO_3_, 85 °C, PEG200	90	9	75	21
Fe_3_O_4_@SiO_2_–NHC–Pd(ii) (0.43)	✗	1.5 h, piperidine, 90 °C, solvent-free	95	8	147.3	22
MNP-CD-Pd (0.075)	✗	6 h, K_2_CO_3_, 100 °C, H_2_O/DMF	96	5	213	23
Pd-TPOP-1 (5)	✗	8 h, hexamine, 100 °C, DMF	70	4	1.75	24
Fe_3_O_4_@SiO_2_/Schiff base/Pd(ii) (0.5)	✗	1 h, K_2_CO_3_, 90 °C, DMF	93	6	186	25
CPS-MNPs-NNN-Pd (0.5)	✗	7 h, K_2_CO_3_, 90 °C, H_2_O/DMF	91	5	26	26
Pd/MgLa (1.5)	✗	10 h, Et_3_N, 80 °C, DMF	90	3	6	27
Pd(0)/Cu^2+^@MMT/CS (1)	✓	8 h, Na_2_CO_3_, 80 °C, H_2_O/DME	96	6	12	28
MgO@PdCu (0.05)	✓	24 h, DABCO, 60 °C, DMF	97	8	80.8	29
Pd(ii)-PMO-P-2 (0.3)	✗	5 h, Et_3_N, 60 °C, H_2_O	96	7	64	30
Pd@PANI (0.005)	✗	48 h, Et_3_N, 80 °C, MeCN	86	6	358	31
SBA-15-TAT-Pd(ii) (0.62)	✗	1 h, Et_3_N, 120 °C, DMF	90	5	145	32
Tetraimine Pd(0) complex (0.4)	✗	0.75 h, K_2_CO_3_, 100 °C, DMF	94	6	313	33
Pd–CoFe_2_O_4_ MNPs (5)	✗	6 h, K_2_CO_3_, 70 °C, EtOH	90	5	3	34
Pd/Nf-G (0.3)	✗	6 h, K_2_CO_3_, 78 °C, EtOH	97	5	53.8	35
Pd/SNW1 (1.29)	✗	2 h, pyrrolidine, 70 °C, H_2_O	98	5	38	36
Pd@PTC-POP (0.3)	✗	1 h, Et_3_N, 100 °C, H_2_O	96	5	320	**This work**
	✗	2 h, Et_3_N, 100 °C, H_2_O	99	5	165	

## Experimental

### Materials and measurements

All chemicals and solvents were at least of analytic grade and employed as received without further purification. The elemental analysis was conducted on a PerkinElmer Model 2400 analyser. MS spectra were obtained by Bruker maxis ultra-high resolution-TOF MS system. NMR data were collected using an AM-400 spectrometer. The solid-state NMR spectra were obtained on Agilent 600 DD2 spectrometer. Infrared spectra were obtained in the 400–4000 cm^−1^ range using a Bruker ALPHA FT-IR spectrometer. Powder X-ray diffraction (PXRD) measurements were performed at 293 K on a D8 ADVANCE diffractometer (Cu Kα, *λ* = 1.5406 Å). ICP analysis was performed on an IRIS InterpidII XSP and NU AttoM. XPS spectra were obtained from PHI Versaprobe II. Thermogravimetric analyses were carried out on a TA Instrument Q5 simultaneous TGA under flowing nitrogen at a heating rate of 10 °C min^−1^. HRTEM (high resolution transmission electron microscopy) analysis was performed on a JEOL 2100 Electron Microscope at an operating voltage of 200 kV. The scanning electron microscopy (SEM) micrographs were recorded on a Gemini Zeiss Supra TM scanning electron microscope equipped with energy-dispersive X-ray detector (EDX).

### Synthesis of 4,7-dibromo-1-ethyl-1*H*-benzo[*d*]imidazole

A mixture of 4,7-dibromo-2,1,3-benzothidiazole (5.86 g, 20 mmol) and CoCl_2_·6H_2_O (48 mg, 0.2 mmol, 1 mol%) in EtOH/THF (110 mL/50 mL) was refluxed for 3 h. Then, NaBH_4_ (2.27 g, 60 mmol) was carefully added in several portions. After that, the mixture was refluxed for additional 6 h. After addition of water (80 mL), the precipitate was filtered through Celite. The organic solvent was removed under vacuum and extracted with dichloromethane (3 × 80 mL). The combined organic layer was washed with brine and dried with anhydrous MgSO_4_. The crude product was purified by column chromatography (eluent: petroleum ether/EtOAc = 5/1) to give 3,6-dibromobenzene-1,2-diamine as a yellow solid. ^1^H NMR (400 MHz, CDCl_3_) *δ* 6.84 (s, 1H), 3.89 (s, 2H).

A mixture of 3,6-dibromobenzene-1,2-diamine (4.79 g, 18.0 mmol), HC(OEt)_3_ (3.89 mL, 23.5 mmol) and NH_2_SO_3_H (95 mg, 0.98 mmol) was stirred overnight at room temperature to afford 4,7-dibromo-1*H*-benzo[*d*]imidazole as a yellow solid (3.92 g, 71%). ^1^H NMR (400 MHz, DMSO-*d*^6^) *δ* 8.80 (s, 1H), 7.48 (s, 2H). HRMS (ESI-TOF) calcd for C_7_H_4_Br_2_N_2_ ([M + H]^+^) 276.8729, found 276.8757.

A mixture of 4,7-dibromo-1*H*-benzo[*d*]imidazole (552 mg, 2 mmol), K_2_CO_3_ (0.83 g, 6 mmol) in anhydrous ethanol (15 mL) was heated to reflux. Then, iodoethane (0.32 mL, 4 mmol) was added dropwise. After refluxed for additional 8 h, the crude product was purified by column chromatography (eluent: petroleum ether/EtOAc = 10/1) to give the product as a bright yellow oil. 4,7-Dibromo-1-ethyl-1*H*-benzo[*d*]imidazole (0.6 g, 98%). ^1^H NMR (400 MHz, CDCl_3_) *δ* 7.96 (s, 1H), 7.31 (d, *J* = 4.2 Hz, 2H), 4.57 (q, *J* = 7.2 Hz, 2H), 1.55 (t, *J* = 7.2 Hz, 3H).; ^13^C NMR (101 MHz, CDCl_3_) *δ* 144.9, 128.3 (2C), 126.2 (2C), 113.5, 102.2, 41.7, 17.7. IR (KBr): 3402 (s), 3076 (m), 2980 (s), 2931 (m), 1600 (m), 1497 (vs), 1475 (m), 1461 (m), 1373 (s), 1336 (s), 1327 (s), 1271 (m), 1257 (w), 1211 (m), 1184 (w), 1110 (vs), 917 (s), 893 (m), 796 (m), 701 (w), 634 (m). HRMS (ESI-TOF) calcd for C_9_H_8_Br_2_N_2_ ([M + H]^+^) 304.9042, found 304.9083.

### Synthesis of A

A mixture of 4,7-dibromo-1-ethyl-1*H*-benzo[*d*]imidazole (1.21 g, 4 mmol), 1-bromododecane (2.49 g, 10 mmol) in of acetonitrile (4 mL) was heated to 110 °C for 16 h. The solvent was removed under vacuum and the solid was completely washed with ethyl acetate and Et_2_O and dried in air to afford a milky powdered imidazolium salt A (1.24 g, 56%). ^1^H NMR (400 MHz, CDCl_3_) *δ* 11.87 (s, 1H), 7.65 (s, 2H), 5.08 (q, *J* = 7.0 Hz, 2H), 5.01–4.91 (m, 2H), 2.13–2.00 (m, 2H), 1.77 (t, *J* = 7.0 Hz, 3H), 1.54–1.42 (m, 2H), 1.36 (dd, *J* = 13.4, 6.6 Hz, 2H), 1.27 (m, *J* = 19.0 Hz, 14H), 0.88 (t, *J* = 6.8 Hz, 3H). ^13^C NMR (101 MHz, CDCl_3_) *δ* 146.5, 132.9, 132.8, 130.7, 130.7, 105.8, 105.8, 49.8, 45.4, 31.9, 31.8, 29.6 (2C), 29.5, 29.4, 29.3, 29.1, 26.2, 22.7, 17.2, 14.2.; IR (KBr): 3364 (s), 3139 (w), 2957 (m), 2920 (vs), 2851 (s), 1602 9 (m), 1568 (s), 1464 (s), 1390 (s), 1366 (s), 1329 (w), 1251 (w), 1227 (m), 1186 (w), 1138 (m), 1121 (w), 1089 (m), 1040 (s), 898 (w), 835 (m), 721 (w), 623 (m). HRMS (ESI-TOF) calcd for C_21_H_33_Br_2_N_2_^+^ ([M]^+^) 473.0985, found 473.0971.

### Synthesis of B

SiCl_4_ was added dropwise to an absolute ethanol (60 mL) solution of 4-bromoacetophenone (5.97 g, 30 mmol) at 0 °C under N_2_ atmosphere. After stirred at 0 °C for 1 h, the mixture was stirred for additional 24 h at room temperature. After addition of 100 mL of water, the reaction system was extracted with dichloromethane (3 × 100 mL). The organic phase was dried with anhydrous MgSO_4_ and filtered. The solvent was removed under vacuum and recrystallized from ethanol to afford 1,3,5-tris(4-bromophenyl)benzene as a white solid (4.4 g, 81%). ^1^H NMR (400 MHz, CDCl_3_) *δ* 7.72 (s, 3H), 7.63 (d, *J* = 8.4 Hz, 6H), 7.56 (d, *J* = 8.4 Hz, 6H). ^13^C NMR (101 MHz, CDCl_3_) *δ* 141.5 (3C), 139.6 (3C), 132.1 (6C), 128.9 (6C), 125.0 (3C), 122.1 (3C).

A mixture of 1,3,5-tris(4-bromophenyl)benzene (2.17 g, 4 mmol), bis(pinacolato)diboron (4.6 g, 18 mmol), KOAc (5.88 g, 60 mmol) and Pd(dppf)Cl_2_ (0.58 g, 0.8 mmol) in 50 mL of DMF was heated at 80 °C for 8 h under N_2_ atmosphere. After additional of 50 mL of water, the reaction system was extracted with ethyl acetate (3 × 50 mL). The combined organic layer was dried with anhydrous MgSO_4_, filtered and concentrated. The product was purified by column chromatography (eluent: petroleum ether/EtOAc = 50/1) to afford 1,3,5-tri(4-pinacholatoborolanephenyl)benzene (B) as a white solid (2.46 g, 90%). ^1^H NMR (400 MHz, CDCl_3_): *δ* 7.93 (d, *J* = 8.0 Hz, 6H), 7.82 (s, 3H), 7.71 (d, *J* = 8.0 Hz, 6H), 1.37 (s, 36H). ^13^C NMR (101 MHz, CDCl_3_) *δ* 143.7 (3C), 142.3 (3C), 135.4 (6C), 126.7 (6C), 125.6 (6C), 83.9 (6C), 24.9 (12C). IR (KBr): 2978 (vs), 2931 (m), 1610 (m), 1553 (w), 1443 (m), 1389 (m), 1370 (vs), 1360 (s), 1321 (m), 1288 (vs), 1205 (m), 1188 (m), 1175 (s), 1126 (vs), 1020 (w), 960 (m), 849 (s), 798 (w), 744 (m), 660 (m), 578 (w), 547 (m). HRMS (ESI-TOF) calcd for C_42_H_51_B_3_O_6_ ([M + H]^+^) 685.4045, found 685.4063.

### Synthesis of Pd@PTC-POP

A mixture of A (664 mg, 1.2 mmol), B (550 mg, 0.8 mmol) and Pd(PPh_3_)_4_ (0.14 g, 0.12 mmol) in DMF (120 mL) and K_2_CO_3_ aqueous solution (10 mL, 2 M) was heated at 110 °C for 72 h in N_2_. After cooled to room temperature, the obtained crude product was completely washed with DMF, H_2_O and MeOH, respectively. The resulted solids were further Soxhlet extracted with dichloromethane and then dried at 110 °C *in vacuo* to afford Pd@PTC-POP as dark gray solids (0.32 g, 45%). IR (KBr): 3390 (s), 3030 (w), 2924 (vs), 2852 (s), 2041 (w), 1705 (s), 1595 (m), 1518 (w), 1492 (m), 1463 (s), 1377 (m), 1225 (m), 1085 (m), 1015 (m), 830 (m), 749 (w), 616 (w). Anal. calcd: C, 66.54; H, 6.65; N, 4.25; Br, 15.3; Pd, 0.547 wt% (Pd wt% was determined by ICP).

### General procedure for the model Sonogashira cross-coupling reaction between iodobenzene and phenylacetylene

A mixture of iodobenzene (0.5 mmol, 56 μL), phenylacetylene (1.0 mmol, 110 μL), Et_3_N (1.5 mmol, 210 μL) and Pd@PTC-POP (29 mg, 0.3 mol% Pd equiv) in 2 mL H_2_O was stirred at 100 °C for 2 h in air. After addition of water (10 mL), the mixture was extracted with ethyl acetate (3 × 80 mL). The organic phase was dried over anhydrous MgSO_4_ and concentrated in vacuum. The residue was purified by column chromatography on silica gel using hexane as eluent to give the product as white solid (89 mg, 99%).

### General procedures for the recycle of Pd@PTC-POP

After each catalytic run, the solid catalyst was retrieved by centrifugation, washed with EtOH (3 × 2 mL), CH_2_Cl_2_ (3 × 2 mL), and dried at 110 °C for 2 h and then was reused for the next catalytic run under the same reaction conditions.

### Preparation of Pd-free PTC-POP and its catalytic activity

A mixture of Pd@PTC-POP (100 mg) and HNO_3_ (5 mL, 16 mol L^−1^) was stirred at room temperature for 24 h. Then, HNO_3_ was removed by centrifugation, and the obtained solid was washed with HNO_3_ (3 × 2 mL), H_2_O (3 × 2 mL), dried at 110 °C to give PTC-POP as a dark yellow solid (95 mg). The Pd content was 0.021 wt%, which was determined by ICP analysis. The obtained Pd-free PTC-POP (29 mg) was used as the catalyst to promote the model Sonogashira coupling reaction between iodobenzene and phenylacetylene, and no desired product was detected (Fig. S3, ESI†).

### Preparation of Pd@PTC-POP (I^−^) and its catalytic activity

Pd@PTC-POP (I^−^) was obtained by anion exchange. Pd@PTC-POP (Br^−^) (0.1 g) was stirred in 10 mL of saturated ethanol solution of the potassium iodide for 24 h, the resulting precipitate was washed with ethanol (5 mL) several times to afford Pd@PTC-POP (I^−^). The I and Pd amounts were determined as 0.333 and 0.508 wt%, respectively. No Br species was detected.

A mixture of iodobenzene (0.5 mmol, 56 μL), phenylacetylene (1.0 mmol, 110 μL), Et_3_N (1.5 mmol, 210 μL) and Pd@PTC-POP (I^−^) (31 mg, 0.3 mol% Pd equiv) in 2 mL H_2_O was stirred at 100 °C for 2 h in air. After addition of water (10 mL), the mixture was extracted with ethyl acetate (3 × 10 mL). The organic phase was dried over anhydrous MgSO_4_ and concentrated in vacuum. The residue was purified by column chromatography on silica gel using hexane as eluent to give the coupling product as white solid (89 mg, 99%).

### Synthesis of Pd@POP

A mixture of 4,7-dibromo-1-ethyl-1*H*-benzo[*d*]imidazole (365 mg, 1.2 mmol), B (550 mg, 0.8 mmol) and Pd(PPh_3_)_4_ (0.14 g, 0.12 mmol) in DMF (120 mL) and K_2_CO_3_ aqueous solution (10 mL, 2 M) was heated at 110 °C for 72 h in N_2_. After cooled to room temperature, the obtained crude product was completely washed with DMF, H_2_O and MeOH, respectively. The resulted solids were further Soxhlet extracted with dichloromethane and then dried at 110 °C *in vacuo* to afford Pd@POP as light gray solids (0.33 g, 79%). IR (KBr): 3649 (w), 3629 (w), 3028 (s), 2980 (m), 1594 (s), 1516 (s), 1492 (vs), 1463 (m), 1448 (m), 1378 (m), 1350 (m), 1243 (s), 1209 (m), 1174 (w), 1081 (m), 1017 (m), 956 (w), 931 (w), 910 (w), 885 (w), 813 (vs), 763 (w), 746 (m), 706 (m), 636 (w), 616 (w), 528 (w). Anal. calcd: C, 75.92; H, 5.06; N, 6.25; Pd, 1.313% (Pd wt% was determined by ICP). The characterization of Pd@POP was shown in Fig. S2, ESI.†

### Catalytic activity of Pd@POP

Catalytic activity of Pd@POP for the model Sonogashira cross-coupling between iodobenzene and phenylacetylene in water was examined under the optimized conditions. The isolated yield for the desired diphenylacetylene was only 33%, indicating that the attached IM-IL species in Pd@PTC-POP indeed played a key role in this Pd-catalysed PTC process.

## Conclusions

In conclusion, we reported a Pd NP loaded and surfactant imidazolium-based ionic liquid (IM-IL) decorated organic polymer Pd@PTC-POP, in which the Pd NP was *in situ* generated during the POP formation *via* Pd-catalysed Suzuki–Miyaura cross-coupling reaction. The generated Pd@PTC-POP can be a highly active phase-transfer catalyst to promote the Sonogashira cross-coupling reactions in aqueous phase especially for the iodo-substituted aromatic substrates. Compared to the reported solid Pd-catalysts, it met the green synthesis and sustainable requirements such as pure water reaction medium, cyclic utilization and high catalytic efficiency. We expect the presented approach to be viable for the construction of many more new POP-based phase-transfer catalytic systems for various organic transformations in water.

## Conflicts of interest

There are no conflicts to declare.

## Supplementary Material

RA-009-C9RA04103F-s001
