# Supplementary material for: Porous organic polymer with in situ generated palladium nanoparticles as a phase-transfer catalyst for Sonogashira cross-coupling reaction in water
Source: RSC Adv. 2019 Jul 12;9(38):21671–8. doi: 10.1039/c9ra04103f (PMC9066429; doi:10.1039/c9ra04103f)
Supplement: RA-009-C9RA04103F-s001 [file RA-009-C9RA04103F-s001.pdf]

Electronic Supplementary Material (ESI) for RSC Advances.
This journal is © The Royal Society of Chemistry 2019

## Supporting Information for

# Porous organic polymer with in situ generated palladium nanoparticle as a phase-transfer catalyst for Sonogashira cross-coupling reaction in water

Ying Dong, *† Yun-Qi Chen,† Jing-Jing Jv, Yue Li, Wen-Han Li, Yu-Bin Dong*

College of Chemistry, Chemical Engineering and Materials Science, Collaborative Innovation Center of Functionalized Probes for Chemical Imaging in Universities of Shandong, Key Laboratory of Molecular and Nano Probes, Ministry of Education, Shandong Normal University, Jinan 250014, P. R. China.

E-mail:
dongyinggreat@163.com
yubindong@sdnu.edu.cn

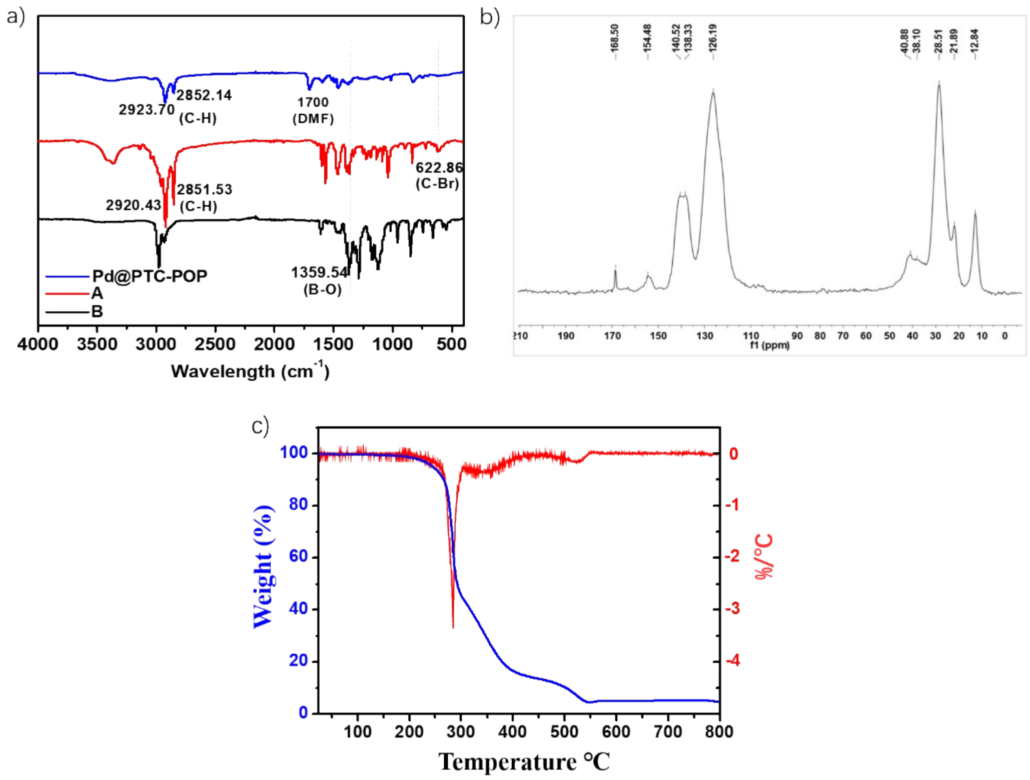


**Fig. S1** IR spectrum of **Pd@PTC-POP**. Solid-state $^{13}$C NMR of **Pd@PTC-POP**. The peak at 1700 cm$^{-1}$ should be ascribed to the residual DMF in the POP during preparation process, which was further confirmed by the solid-state $^{13}$C NMR (ca. 169 ppm) and the TGA analysis (ca. 3% weight loss below 220°C).

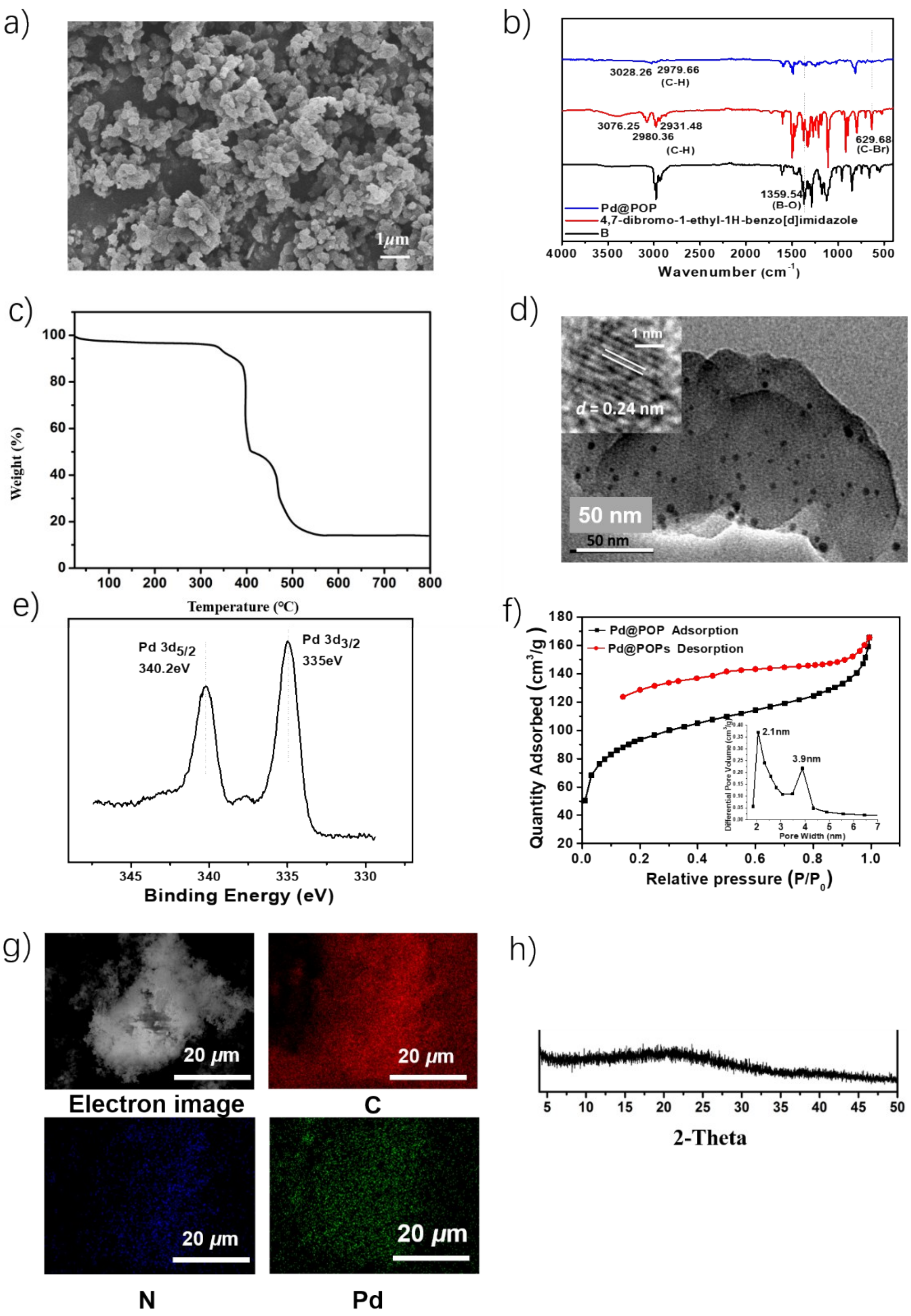


**Fig. S2** a) SEM image of **Pd@POP**. It was obtained as the irregular granular particles. b) IR spectra of **Pd@POP** and its precursors. It showed that the characteristic peaks at ca.1359 $cm^{-1}$ and 629 $cm^{-1}$ for the B-O and C-Br species significantly decreased after the coupling reaction, meanwhile the bands at 3028 and 2979 $cm^{-1}$ for the alkyl groups appeared, indicating the formation of **Pd@PTC-POP**. c) TGA trace of **Pd@POP**. No significant weight loss was observed before ca. 300 °C. d) HRTEM image of **Pd@POP**. The Pd NPs (2–6 nm) were homogeneously distributed in the POP matrix, and the atomic lattice fringes with an interplanar spacing of 0.24 nm for face-centred cubic (fcc) Pd NPs were clearly observed. e) XPS spectrum of **Pd@POP**, indicating the valence of Pd species in the POP is zero. f) $N_2$ sorption isotherm of **Pd@POP**. Its surface area is 331.9 $m^2$ $g^{-1}$. Its pore size distribution plot is shown as the inset. g) SEM-EDX spectrum of **Pd@POP**. h) PXRD pattern of **Pd@POP**, suggesting its amorphous nature.

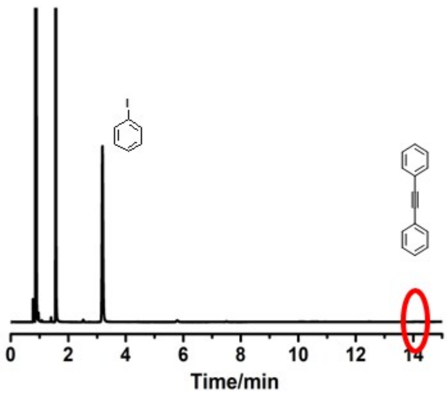


**Fig. S3** GC analysis for the model Sonogashira coupling reaction between iodobenzene and phenylacetylene catalyzed by **PTC-POP** (for Table 1, entry 11).

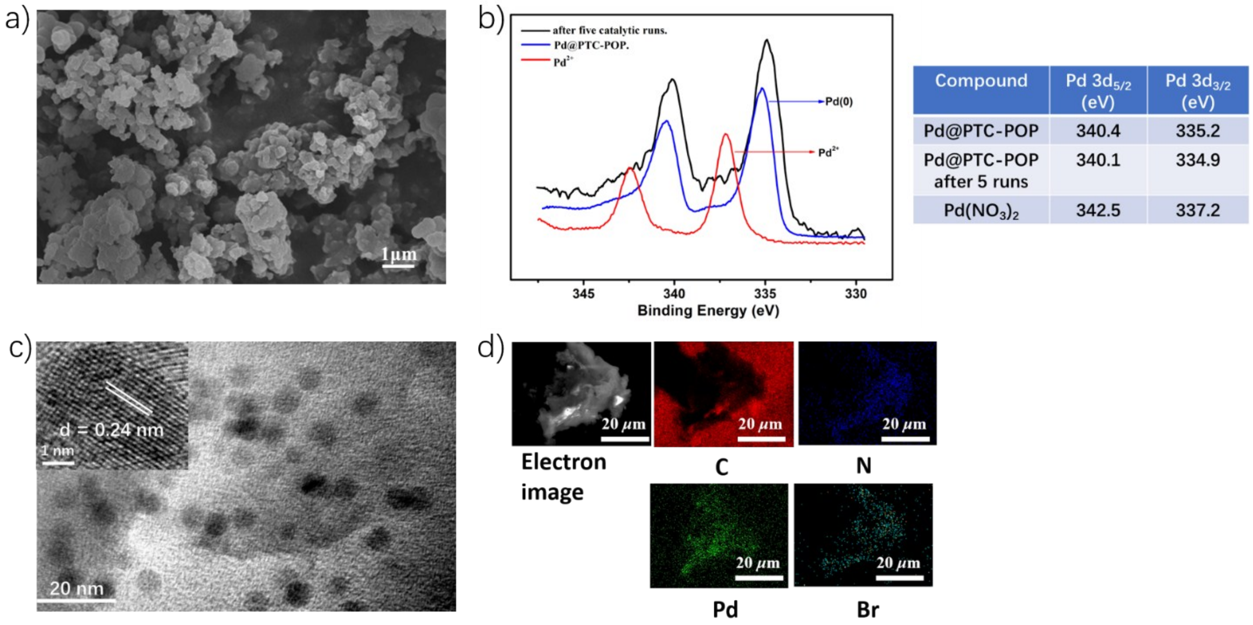


| Compound | Pd $3d_{5/2}$ (eV) | Pd $3d_{3/2}$ (eV) |
|---|---|---|
| Pd@PTC-POP | 340.4 | 335.2 |
| Pd@PTC-POP after 5 runs | 340.1 | 334.9 |
| $Pd(NO_3)_2$ | 342.5 | 337.2 |

**Fig. S4** SEM (a), XPS (b), HRTEM (c) and SEM-EDX (d) spectra of the **Pd@PTC-POP** after five catalytic runs.

**Product characterization (for Table 2)**

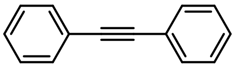

**3a:** $^1$H NMR (400 MHz, $CDCl_3$) δ 7.57 (dd, *J* = 7.3, 2.3 Hz, 4H), 7.46-7.31 (m, 6H).; $^{13}$C NMR (101 MHz, $CDCl_3$) δ 131.6 (4C), 128.4 (4C), 128.3 (2C), 123.3 (2C), 89.4 (2C).; HRMS (ESI-TOF) calcd for $C_{14}H_{10}$ ([M+H]$^+$) 179.0863, found 179.0891.

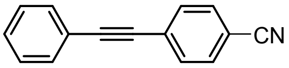


**3b:** 1H NMR (400 MHz, $CDCl_3$) δ 7.66-7.62 (m, 2H), 7.62-7.58 (m, 2H), 7.55 (dd, *J* = 6.5, 3.2 Hz, 2H), 7.39 (d, *J* = 2.2 Hz, 2H), 7.37 (d, *J* = 1.4 Hz, 1H).; $^{13}$C NMR (101 MHz, $CDCl_3$) δ 132.1 (2C), 132.1 (2C), 131.8 (2C), 129.2, 128.5 (2C), 128.3, 122.2, 118.6, 111.5, 93.8, 87.7.; HRMS (ESI-TOF) calcd for $C_{15}H_9N$ ([M+Na]$^+$) 226.0635, found 226.0622.

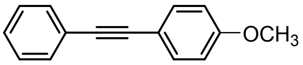


**3c and 3i:** $^{1}H$ NMR (400 MHz, $CDCl_3$) δ 7.55 (d, *J* = 2.0 Hz, 1H), 7.53 (d, *J* = 1.5 Hz, 1H), 7.52 (s, 1H), 7.49 (s, 1H), 7.40-7.33 (m, 3H), 6.92 (s, 1H), 6.90 (s, 1H), 3.86 (s, 3H).; $^{13}C$ NMR (101 MHz, $CDCl_3$) δ 159.6, 133.1 (2C), 131.5 (2C), 128.3 (2C), 128.0, 123.6, 115.4, 114.0 (2C), 89.4, 88.1, 55.3.; HRMS (ESI-TOF) calcd for $C_{15}H_{12}O$ ($[M + Na]^+$) 231.0788, found 231.0766.

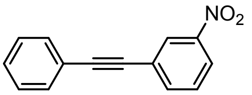


**3d:** $^{1}H$ NMR (400 MHz, $CDCl_3$) δ 8.34-8.28 (m, 1H), 8.11 (ddd, *J* = 8.3, 2.2, 1.0 Hz, 1H), 7.78-7.72 (m, 1H), 7.48 (dt, *J* = 13.0, 5.2 Hz, 3H), 7.31 (dd, *J* = 4.4, 2.3 Hz, 3H).; $^{13}C$ NMR (101 MHz, $CDCl_3$) δ 148.2, 137.2, 131.8 (2C), 129.4, 129.1, 128.5 (2C), 126.4, 125.2, 122.9, 122.2, 91.9, 86.9.; HRMS (ESI-TOF) calcd for $C_{14}H_9NO_2$ ($[M + Na]^+$) 246.0533, found 246.0520.

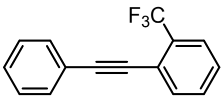


**3e:** $^{1}H$ NMR (400 MHz, $CDCl_3$) δ 7.59 (t, *J* = 7.4 Hz, 2H), 7.50-7.45 (m, 2H), 7.42 (t, *J* = 7.6 Hz, 1H), 7.33 (d, *J* = 7.8 Hz, 1H), 7.30-7.25 (m, 3H).; $^{13}C$ NMR (101 MHz, $CDCl_3$) δ 133.7, 131.7 (2C), 131.4, 128.8, 128.4 (2C), 127.9, 126.7, 125.9, 125.0, 122.7, 122.3, 121.6, 94.9, 85.4, 29.7.; HRMS (ESI-TOF) calcd for $C_{15}H_9F_3$ ($[M+Na]^+$) 269.0556, found 269.0530.

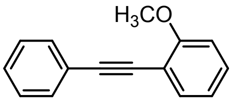


**3f:** $^{1}H$ NMR (400 MHz, CDCl3) δ 7.56 (dd, *J* = 7.3, 2.0 Hz, 2H), 7.50 (dd, *J* = 7.6, 1.5 Hz, 1H), 7.37-7.28 (m, 4H), 6.93 (dd, *J* = 17.3, 8.0 Hz, 2H), 3.92 (s, 3H).; $^{13}C$ NMR (101 MHz, $CDCl_3$) δ 159.9, 133.6, 131.7 (2C), 129.8, 128.3 (2C), 128.1, 123.6, 120.5, 112.4, 110.7, 93.4, 85.7, 55.9.; HRMS (ESI-TOF) calcd for $C_{15}H_{12}O$ ($[M+Na]^+$) 231.0788, found 231.0771.

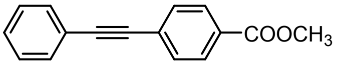


**3g:** $^{1}H$ NMR (400 MHz, $CDCl_3$) δ 8.03 (d, *J* = 1.8 Hz, 1H), 8.02 (d, *J* = 1.8 Hz, 1H), 7.60 (d, *J* = 1.8 Hz, 1H), 7.58 (d, *J* = 1.7 Hz, 1H), 7.57-7.55 (m, 1H), 7.54 (d, *J* = 2.6 Hz, 1H), 7.39-7.35 (m, 3H), 3.93 (s, 3H).; $^{13}C$ NMR (101 MHz, $CDCl_3$) δ 166.6, 131.8 (2C), 131.5 (2C), 129.5 (2C), 129.5, 128.8, 128.5 (2C), 128.0, 122.7, 92.4, 88.7, 52.3.; HRMS (ESI-TOF) calcd for $C_{16}H_{12}O_2$ ($[M+Na]^+$) 259.0737, found 259.0723.

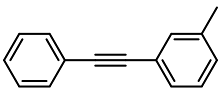

**3h and 3k:** $^{1}H$ NMR (400 MHz, $CDCl_3$) δ 7.58 (dd, *J* = 7.3, 2.1 Hz, 2H), 7.43-7.35 (m, 5H), 7.28 (t, *J* = 7.6 Hz, 1H), 7.19 (d, *J* = 7.6 Hz, 1H), 2.40 (s, 3H).; $^{13}C$ NMR (101 MHz, $CDCl_3$) δ 138.1, 132.2, 131.6 (2C), 129.2, 128.7, 128.4 (2C), 128.3, 128.2, 123.4, 123.1, 89.6, 89.2, 21.3.; HRMS (APCI-TOF) calcd for $C_{15}H_{12}$ ($[M+H]^+$) 193.1019, found 193.1010.

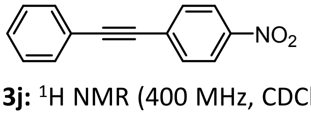


**3j:** $^{1}H$ NMR (400 MHz, $CDCl_3$) δ 8.17-8.15 (m, 1H), 8.15-8.13 (m, 1H), 7.62-7.59 (m, 1H), 7.59-7.57 (m, 1H), 7.49 (dd, *J* = 6.5, 3.2 Hz, 2H), 7.33 (d, *J* = 2.0 Hz, 2H), 7.31 (d, *J* = 1.8 Hz, 1H).; $^{13}C$ NMR (101 MHz, $CDCl_3$) δ 146.0,

131.3 (2C), 130.8 (2C), 129.2, 128.3, 127.5 (2C), 122.6 (2C), 121.1, 93.7, 86.5.; HRMS (ESI-TOF) calcd for $C_{14}H_9NO_2$ ([M+Na]$^+$) 246.0533, found 246.0516.

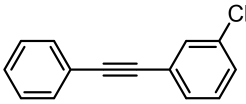


**3l:** $^1$H NMR (400 MHz, $CDCl_3$) δ 7.55-7.48 (m, 3H), 7.39 (dt, *J* = 7.3, 1.3 Hz, 1H), 7.36-7.31 (m, 3H), 7.29 (dt, *J* = 8.0, 1.6 Hz, 1H), 7.25-7.21 (m, 1H). $^{13}$C NMR (101 MHz, $CDCl_3$) δ 134.2, 131.7 (2C), 131.5, 129.8, 129.6, 128.7, 128.6, 128.5 (2C), 125.1, 122.8, 90.6, 88.0.; HRMS (APCI-TOF) calcd for $C_{14}H_9Cl$ ([M+H]$^+$) 213.0473, found 213.0468.

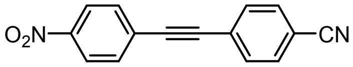


**3m:** $^1$H NMR (400 MHz, $CDCl_3$) δ 8.29 (s, 1H), 8.27 (s, 1H), 7.72 (dd, *J* = 8.5, 4.4 Hz, 4H), 7.67 (d, *J* = 8.4 Hz, 2H).; $^{13}$C NMR (101 MHz, $CDCl_3$) δ 147.5, 132.6 (2C), 132.3 (2C), 132.2 (2C), 129.0, 126.9, 123. 8 (2C), 118.2, 112.6, 92.3, 91.3.; HRMS (ESI-TOF) calcd for $C_{15}H_8N_2O_2$ ([M+Na]$^+$) 271.0486, found 271.0469.

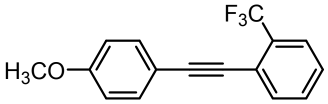


**3n:** $^1$H NMR (400 MHz, $CDCl_3$) δ 7.65 (dd, *J* = 12.4, 7.8 Hz, 2H), 7.53-7.46 (m, 3H), 7.38 (t, *J* = 7.7 Hz, 1H), 6.92-6.89 (m, 1H), 6.89-6.86 (m, 1H), 3.83 (s, 3H).; $^{13}$C NMR (101 MHz, $CDCl_3$) δ 160.1, 133.5, 133.2 (2C), 131.4, 131.1, 127.5, 125.8, 125.0, 122.3,121.9, 114.8, 114.1 (2C), 95.1, 84.3, 55.3, 29.7.; HRMS (ESI-TOF) calcd for $C_{16}H_{11}F_3O$ ([M+H]$^+$) 277.0842, found 277.0931.

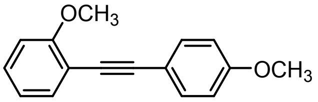


**3o:** $^1$H NMR (400 MHz, $CDCl_3$) δ 7.54-7.46 (m, 3H), 7.32-7.26 (m, 1H), 6.96-6.84 (m, 4H), 3.91 (s, 3H), 3.82 (s, 3H).; $^{13}$C NMR (101 MHz, $CDCl_3$) δ 159.8, 159.5, 133.5, 133.1 (2C), 129.5, 120.5, 115.7, 113.9 (2C), 112.7, 110.7, 93.5, 84.3, 55.9, 55.3.; HRMS (ESI-TOF) calcd for $C_{16}H_{14}O_2$ ([M+Na]$^+$) 261.0894, found 261.0876.

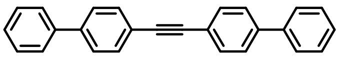

**3p:** $^1$H NMR (400 MHz, $CDCl_3$) δ 7.65-7.49 (m, 5H), 7.49-7.31 (m, 4H).; $^{13}$C NMR (101 MHz, $CDCl_3$) δ 141.0, 140.4, 132.1 (4C), 131.6 (4C), 128.8 (4C), 128.4 (4C), 128.3 (2C), 127.6 (2C), 123.4, 122.2, 90.1, 89.3.; HRMS (APCI-TOF) calcd for $C_{26}H_{18}$ ([M+H]$^+$) 331.1489, found 331.1481.
